# Increased Cortical Inhibition in Autism-Linked Neuroligin-3R451C Mice Is Due in Part to Loss of Endocannabinoid Signaling

**DOI:** 10.1371/journal.pone.0140638

**Published:** 2015-10-15

**Authors:** Haley E. Speed, Irene Masiulis, Jay R. Gibson, Craig M. Powell

**Affiliations:** 1 Department of Neurology and Neurotherapeutics, University of Texas Southwestern Medical Center, Dallas, Texas, United States of America; 2 Quantitative Morphology Core, University of Texas Southwestern Medical Center, Dallas, Texas, United States of America; 3 Department of Neuroscience, University of Texas Southwestern Medical Center, Dallas, Texas, United States of America; 4 Department of Psychiatry and Neuroscience Graduate Program, University of Texas Southwestern Medical Center, Dallas, Texas, United States of America; Virginia Tech Carilion Research Institute, UNITED STATES

## Abstract

A single, maternally inherited, X-linked point mutation leading to an arginine to cysteine substitution at amino acid 451 (R451C) of Neuroligin 3 (NLGN3^R451C^) is a likely cause of autism in two brothers. Knockin mice expressing the Nlgn3^R451C^ mutation in place of wild-type Nlgn3 demonstrate increased inhibitory synaptic strength in somatosensory cortex, resulting in an excitatory/inhibitory (E/I) imbalance that is potentially relevant for autism-associated behavioral deficits characteristic of these mice. We have replicated the increase in evoked inhibitory postsynaptic currents (eIPSCs) onto layer II/III cortical pyramidal neurons. We also find that increased frequency of spontaneous mIPSCs in Nlgn3^R451C^ mice occurs in the absence of action potential-driven transmission. This suggests the E/I imbalance is due to changes at the synapse level, as opposed to the network level. Next, we use paired whole-cell recordings in an attempt to identify specific interneuron subtypes affected by the Nlgn3^R451C^ mutation. Curiously, we observe no change in the amplitude of cell-to-cell, unitary IPSCs (uIPSCs) from parvalbumin-positive (PV) or somatostatin-positive (SOM) interneurons onto pyramidal neurons. We also observe no change in the number or density of PV and SOM interneurons in LII/III of somatosensory cortex. This effectively rules out a role for these particular interneurons in the increased inhibitory synaptic transmission, pointing to perhaps alternative interneuron subtypes. Lastly, impaired endocannabinoid signaling has been implicated in hippocampal synaptic dysfunction in Nlgn3^R451C^ mice, but has not been investigated at cortical synapses. We find that bath application of the CB1 antagonist, AM 251 in WT mice eliminates the Nlgn3^R451C^ increase in eIPSC amplitude and mIPSC frequency, indicating that increased inhibitory transmission in mutant mice is due, at least in part, to a loss of endocannabinoid signaling through CB1 receptors likely acting at interneurons other than PV or SOM.

## Introduction

Mutations in the genes encoding the Neuroligin family of trans-synaptic cell-adhesion proteins [1–3] and their binding partners the Neurexins [4] are associated with autism. An autism-associated point mutation in Neuroligin 3 (Nlgn3) resulting in an amino acid change from arginine to cysteine at residue 451 (R451C) has been successfully replicated in a genetic mouse model [5]. This mutation results in enhanced spatial learning and memory and impaired social interaction as well as region-specific alterations in the balance of excitatory and inhibitory synaptic transmission (E/I balance) [5–7]. In the hippocampus of Nlgn3^R451C^ mutants, excitatory synaptic transmission is dramatically increased due to increased AMPA receptor-mediated currents and altered NMDA receptor kinetics, but overall, inhibitory synaptic transmission is not affected [6]. Conversely, in somatosensory cortex, excitatory synaptic transmission is normal, but inhibitory synaptic transmission is increased [5].

The mechanisms underlying increased inhibitory transmission in somatosensory cortex have not yet been determined. Tabuchi et al. [5] identified an increase in the input/output (I/O) relationship of stimulus intensity to amplitude of the inhibitory postsynaptic current (IPSC) onto pyramidal neurons in layer II/III of somatosensory cortex [5]. In the same study, the frequency of spontaneous inhibitory transmission onto LII/III pyramidal neurons was increased, and staining for the inhibitory marker VGAT demonstrated an increased number of putative inhibitory synaptic puncta in Nlgn3^R451C^ mice [5]. Taken together, these findings suggested an increase in inhibitory synapse number as a possible explanation for the increased cortical inhibitory synaptic transmission. However, there was not a significant increase in the number of symmetric synapses by electron microscopy, suggesting no change in inhibitory synapse number. Also, there was no change in paired-pulse ratio to indicate a change in presynaptic function. Finally, because the frequency of spontaneous events was not measured in the presence of the Na^+^ channel blocker, TTX, the relative contributions of spontaneous network activity and spontaneous GABA release could not be determined, leading to difficulty in interpretation.

We have expanded on these initial studies in an attempt to clarify the mechanisms of increased inhibitory synaptic transmission in Nlgn3^R451C^ mice, hypothesizing that increased cortical inhibitory transmission in Nlgn3^R451C^ mice could be due to: 1) Increased number or strength of interneuron synapses onto pyramidal neurons, 2) Increased number of interneurons, or 3) Impaired tonic endocannabinoid signaling affecting neurotransmitter release probability. First, we replicate the increased eIPSC amplitude. Second, we demonstrate that the increase in sIPSC frequency is independent of network activity in the presence of TTX, demonstrating increased inhibitory synaptic drive at the level of inhibitory synapses onto pyramidal neurons rather than a network or circuit-level effect. Next, we determine the effects of the Nlgn3^R451C^ mutation at synapses between Parvalbumin-positive (PV) and Somatostatin-positive (SOM) interneurons and pyramidal neurons. The Nlgn3^R451C^ mutation has been found to have differential effects on hippocampal inhibitory synaptic transmission with respect to interneuron subtype [8]. Notably, synaptic strength is decreased at PV synapses onto CA1 pyramidal neurons (PV → PYR), while SOM—> PYR synapses are not affected. Likewise, the Neuroligin 2 (Nlgn2) knockout mouse exhibits an E/I imbalance as the result of decreased unitary inhibitory synaptic connections between PV interneurons and pyramidal neurons, but not between SOM interneurons and pyramidal neurons [9]. Expanding on these findings, we have analyzed the effect of the Nlgn3^R451C^ mutation on these two distinct cortical inhibitory networks using paired recordings from connected PV → PYR neuron pairs and SOM → PYR pairs. If the Nlgn3^R451C^ mutation differentially affects PV or SOM interneuron subtypes in LII/III of somatosensory cortex, as well as hippocampus, understanding this selectivity could potentially lead to novel treatment targets for restoration of normal E/I balance.

We also investigated the role of endocannabinoid (eCB) retrograde signaling in the E/I imbalance, as impaired tonic eCB signaling has been reported in hippocampus of Nlgn3^R451C^ mice [8]. eCBs have been well-studied in hippocampus, acting as retrograde signals that bind to presynaptic CB1 receptors, thereby decreasing the probability of GABA release by limiting Ca^2+^ influx through N-type (Cav2.2) channels [10]. However, the role of tonic eCB signaling in LII/III of somatosensory cortex is unknown, and may play a crucial role in both cortex and hippocampus of Nlgn3^R451C^ mice, despite opposite shifts in the E/I balance.

Though we observe no effect of the Nlgn3^R451C^ mutation on either PV → PYR or SOM → PYR reciprocal connectivity or synaptic strength, we do observe decreased sensitivity of inhibitory transmission in general to the CB1 receptor antagonist AM 251 in the cortex of Nlgn3^R451C^ mice compared to WT. We also found that application of AM 251 to LII/III pyramidal neurons in WT mice mimics the increased mIPSC frequency observed in Nlgn3^R451C^ mice and has little or no effect on the Nlgn3^R451C^ mutants. Therefore, loss of eCB signaling is at least contributing in part to the increased inhibitory synaptic transmission in somatosensory cortex of Nlgn3^R451C^ mice, and likely occurs at synapses not involving PV or SOM interneurons.

## Materials and Methods

### Animal models

“Knockin” mice were generated by site-directed mutagenesis of Nlgn3 exon 7 leading to an arginine 451 to cysteine (R451C) substitution in the region of the extracellular acetylcholinesterase-like region [5], and were provided by Thomas Südhof (Stanford University Medical School, Stanford, CA). Nlgn3^R451C^ heterozygous females were mated to wild-type (WT) male mice expressing EGFP in either SOM inhibitory interneurons (GIN mice, Jackson Lab) [11] or PV inhibitory interneurons (G42 crossed with CB6F1/J) kindly provided to JRG by Josh Huang (Cold Spring Harbor Laboratory, Cold Spring Harbor, NY) [12]. Animals were housed in ventilated microisolator cages with unrestricted access to commercial irradiated diet, as well as to purified water through an automated watering system, in a pathogen-free barrier facility. All experiments were performed on Nlgn3^R451C^ and WT littermate pairs (P13-17) on a mixed genetic background by an experimenter blind to genotype.

### Ethics Statement

All experimental procedures were approved by the Institutional Animal Care and Use Committee of the UT Southwestern Medical Center and are in accordance with the National Institutes of Health policy on the care and use of laboratory animals (Animal Protocol Number 0941-07-03-1). All animals underwent deep anesthesia with isoflurane prior to any procedures to minimize suffering.

### Genotyping

Mice were genotyped using the iProof High-Fidelity DNA Polymerase kit (Bio-Rad) or GoTaq Green Hot Start Master Mix (Promega) and a combination of four primers: GFP forward: AAGTTCATCTGCACCACCG (oIMR0872, Jackson Laboratories); GFP reverse: TCCTTGAAGAAGATGGTGCG (oIMR1416, Jackson Laboratories); Nlgn3 forward: TGTACCAGGAATGGGAAGCAG (Integrated DNA Technologies); Nlgn3 reverse: GGTCAGAGCTGTCATTGTCAC (Integrated DNA Technologies) [5]. PCR resulted in three distinct bands: WT Nlgn3 at 250 bp, Nlgn3^R451C^ at 290 bp, and GFP at 173 bp.

### Acute slice preparation

Acute thalamocortical slices were prepared according to standard techniques [13]. Male mice (P13—P17) were briefly anesthetized with isoflurane (Baxter Healthcare), and the brains quickly removed and submerged in ice-cold dissection artificial cerebral spinal fluid (dACSF) containing (in mM): 75 sucrose, 87 NaCl, 3 KCl, 1.25 NaH_2_PO_4_, 7 MgSO_4_, 26 NaHCO_3_, 20 dextrose, and 0.5 CaCl_2_. 300–350 μM thick slices were cut using a vibrating microtome (Vibratome) in ice-cold dACSF then warmed to 34°C for 30 minutes in normal ACSF containing (in mM): 126 NaCl, 3 KCl, 1.25 NaH_2_PO_4_, 1 MgSO_4_, 26 NaHCO_3_, 25 dextrose, and 2 CaCl_2_. Slices were slowly cooled over a 45 min period to room temperature prior to recording. All experiments were performed at 25 ± 0.5°C in normal ACSF. All solutions were pH 7.4 and saturated with 95% O_2_ / 5% CO_2_.

### Whole-Cell Electrophysiology

Neurons were visualized under fluorescence (for GFP-positive interneurons) or DIC microscopy (for pyramidal neurons) using an AxioExminer D1 (Zeiss) microscope. Immediately after break-in, each cell type was confirmed according to its characteristic resting and firing properties [14–16]. Data were sampled at 10 kHz and filtered at 1–3 kHz using dual Multiclamp 700B amplifiers, a Digidata 1440 digitizer, and Clampex acquisition software (version 10.0, Molecular Devices). Only experiments with a high seal resistance (> 3 GΩ) and access resistance < 25 MΩ were analyzed. Recordings were rejected if the series (R_s_) or input resistance (R_input_) changed by more than 25% or if R_s_ exceeded 25 MΩ during the recording. Capacitance (Cm = Q/V) and input resistance (R = V/I) were measured in response to a hyperpolarizing 10 mV step from resting membrane potential. Similarly, firing properties were determined by a series of 25 pA current steps from resting membrane potential. Internal recording solution contained (in mM): 79 K-Gluconate, 44 KCl, 6 NaCl, 10 HEPES, 0.2 EGTA, 4 ATP-Mg, 0.3 GTP-Na, and 10 sucrose. For I/O curves, 3 mM QX 314 was added to the internal pipette solution to prevent generation of postsynaptic action potentials. Pipette solution was adjusted to pH 7.25 and 290–295 mOsm with a junction potential of ~9 mV. Series resistance and junction potential were not compensated.

For I/O curves, eIPSCs were evoked from LII/III pyramidal neurons at -65 mV holding potential in the presence of 20 μM NBQX and 50 μM D-APV beginning 3–5 min after break-in. The stimulating electrode was placed in LII/III ~150–200 μm lateral to the recorded neuron. mIPSCs were recorded from LII/III pyramidal neurons under similar conditions, but without external stimulation and with the addition of 1 μM TTX to block action potential-dependent synaptic transmission.

uIPSCs were evoked between connected pairs of neurons by 20 Hz trains of 5 presynaptic stimuli generated by an 8 ms depolarizing voltage step (15–25 mV). 15–20 consecutive traces (including failures) were averaged prior to analysis and cell pairs were considered connected if the average peak amplitude of any uIPSC in the train was > 2 pA (including failures). Short-term plasticity (STP) was analyzed by calculating the STP index of uIPSC5 / uIPSC1.

Raw traces were analyzed using ClampFit (version 10.0, Molecular Devices) and statistics were performed using Statistica (version 5.5; StatSoft, Inc) and Prism GraphPad (version 5, GraphPad Software). Data are presented as mean ± standard error of the mean (SEM) except where indicated. Sample size (*n*) represents number of cells or connected pairs of cells from a minimum of 6 mice per group, unless otherwise stated. Pairwise comparisons were made using an unpaired, two-tailed Student’s *t*-test unless otherwise specified. Significance was determined as *P* < 0.05.

### Compounds

TTX (Octahydro—12- (hydroxymethyl)-2-imino-5,9:7,10a-dimethano-10aH-[1,3]dioxocino[6,5-d]pyrimidine-4,7,10,11,12-pentol), NBQX (2,3-Dioxo-6-nitro-1,2,3,4-tetrahydrobenzo[*f*]quinoxaline-7-sulfonamide), D-APV (D-(-)-2-Amino-5-phosphonopentanoic acid), AM 251 (N-(Piperidin-1-yl)-5-(4-iodophenyl)-1-(2,4-dichlorophenyl)-4-methyl-1H-pyrazole-3-carboxamide), ACEA (Arachidonyl-2'-chloroethylamide), and QX 314 (N-(2,6-Dimethylphenylcarbamoylmethyl) triethylammonium bromide) were obtained from Tocris Bioscience. Phosphate buffered saline (PBS) was obtained from BioRad and paraformaldehyde (PFA) was obtained from Sigma-Aldrich. All other chemicals and compounds were obtained from Fisher Scientific.

### Tissue preparation and immunohistochemistry

Male mice (P13—P17) were briefly anesthetized with isoflurane (Baxter Healthcare), and the brains quickly removed. Brains were briefly washed in 1X PBS then transferred to a solution containing 4% PFA in 1X PBS. After 36 hrs, brains were transferred to a solution containing 30% sucrose and 1% NaN_3_ in 1X PBS. Brains were sectioned coronally in a 1:10 series of 30 μm thick sections on a freezing microtome. Brain sections were stained for either somatostatin or parvalbumin with a Nissl counterstain. Sections from one well of the 1:10 series were mounted onto charged slides for each of the stains and allowed to dry for one hour. Only those slides stained for somatostatin underwent antigen unmasking in 0.01 M citric acid buffer. Otherwise, all slides were blocked in 3% NDS, 0.3% Triton X 100 in PBS and probed with the either a somatostatin (Peninsula Laboratories (BACHEM) T-4546, rabbit, 1:4,000) or a parvalbumin (Swant, PV25, rabbit, 1:10,000) antibody overnight at room temperature. A biotinylated donkey anti-rabbit secondary antibody (Jackson Immunoresearch, 1:200) was used followed by quenching of endogenous peroxidases using 0.3% H_2_O_2_ and amplification using an ABC kit (Vector Laboratories). Staining was visualized using DAB (ThermoScientific) and counterstained using a cresyl violet stain. Slides were dehydrated and coverslipped using DPX mounting medium (Sigma-Aldrich).

### Stereology and Imaging

Unbiased stereological estimation of total PV and SOM cells in the somatosensory cortex of WT and Nlgn3^R451C^ mice was acquired using the optical fractionator approach and the Stereo Investigator software (MicroBrightField, Inc.) equipped with an Olympus BX51 microscope. Boundaries of the entire somatosensory cortex were determined using anatomical landmarks, the ALLEN Mouse Brain Reference Atlas Version 2 (2011), and Paxinos and Franklin Mouse Brain Atlas (Second Edition) and traced at low magnification (2x, NA 0.05). A sampling grid of 700 μm x 700 μm, counting frame of 100 μm x 100 μm, and dissector height of 10 μm (with 1–3 μm guard zones) were used to estimate the number of positively stained cells in a 1:10 series for each animal at high magnification (40x, NA 0.75). The Gundersen coefficient of error (m = 0) was below 0.1 for all animals (*n* = 10 for both WT and Nlgn3^R451C^ groups) (Gundersen and Jensen, 1987). Data are shown as % of the WT average cell number for either PV neurons or SOM neurons in Nlgn3^R451C^ mice (mean ± SEM). Representative images were taken using the 40x objective on the same Olympus BX51 microscope used for stereology.

## Results

### Basal inhibitory synaptic transmission onto LII/III pyramidal neurons is increased in Nlgn3^R451C^ mice

**Fig 1:**
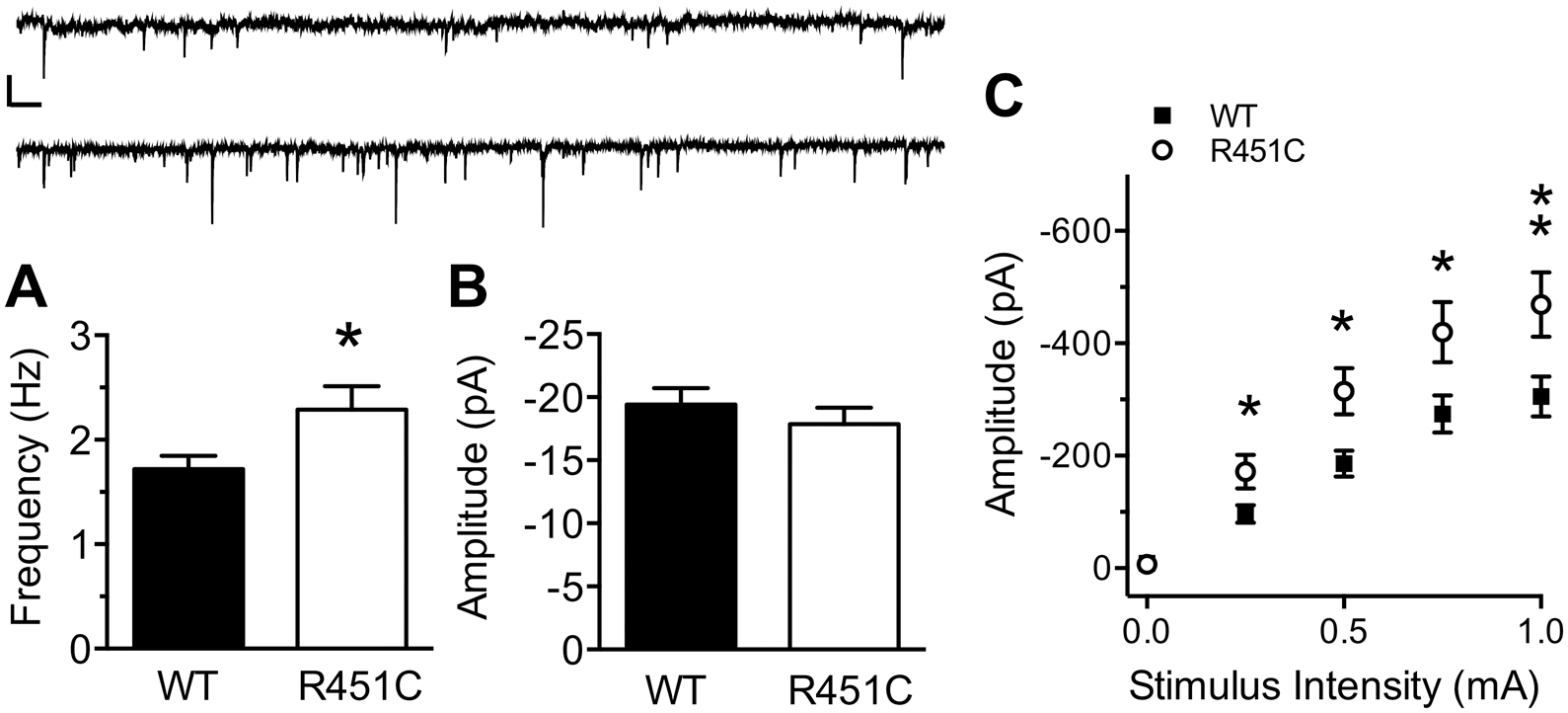
Increased inhibitory synaptic transmission at LII/III synapses from Nlgn3^R451C^ mice. mIPSC frequency (A), but not amplitude (B) of spontaneous inhibitory transmission in the presence of 1 μM TTX is increased Nlgn3^R451C^ mice (WT = 39, Nlgn3^R451C^ = 32). Inset: 15s raw trace from a WT (top) and Nlgn3^R451C^ (bottom) mouse. Scale bar: 25 pA; 0.5 s. C) The input/output (I/O) relationship of eIPSC amplitude to stimulus intensity is stronger in Nlgn3^R451C^ mice compared to WT controls (WT = 33, Nlgn3^R451C^ = 30). * *P* < 0.05, ** *P* < 0.01.

Tabuchi et al. [5] reported that frequency of spontaneous inhibitory synaptic currents (sIPSCs) onto LII/III pyramidal neurons is increased in Nlgn3^R451C^ mice, and that sIPSC amplitude is not affected. These experiments were performed with action potential-dependent transmission intact, and therefore, could represent a network effect as well as a local synaptic effect. To clarify this discrepancy, we measured spontaneous miniature inhibitory currents (mIPSCs) from LII/III pyramidal neurons in the presence of the sodium channel inhibitor TTX (1 μM; Fig 1A and 1B). mIPSC frequency was increased in Nlgn3^R451C^ mice compared to WT mice (WT: 1.72 ± 0.13, Nlgn3^R451C^: 2.29 ± 0.22; *t* (69) = 2.29, *P* = 0.025), but no difference in mIPSC amplitude was observed between genotypes (*t* (69) = 0.84, *P* = 0.406). These data strongly suggest that the Nlgn3^R451C^ mutation causes alterations at the level of inhibitory synapses onto pyramidal neurons, as opposed to a generalized increase in network activity.

Next, we confirmed the original finding that evoked synaptic strength is increased at inhibitory synapses onto LII/III pyramidal neurons of somatosensory cortex of Nlgn3^R451C^ mice [5]. Using whole-cell patch clamp electrophysiology and extracellular stimulation in LII/III we found that the relationship of eIPSC amplitude to extracellular stimulus intensity (0–1 mA) is greater in pyramidal neurons from Nlgn3^R451C^ mice compared to WT controls (Fig 1C. Two-Way RM ANOVA. Genotype: *F*_(1, 61)_ = 7.03, *P* = 0.01; Stimulus Intensity: *F*_(4, 244)_ = 108, *P* < 0.001, Genotype X Stimulus Intensity: *F*_(4, 244)_ = 4.96 *P* < 0.001). The maximum eIPSC amplitude was 34.9% greater in Nlgn3^R451C^ mice than in WT mice (WT: -305.3 ± 34.86, Nlgn3^R451C^: -469.0 ± 56.87; Bonferoni post-hoc analysis, *P* < 0.01), confirming that evoked inhibitory synaptic strength is more robust in Nlgn3^R451C^ mice than WT mice.

**Fig 2:**
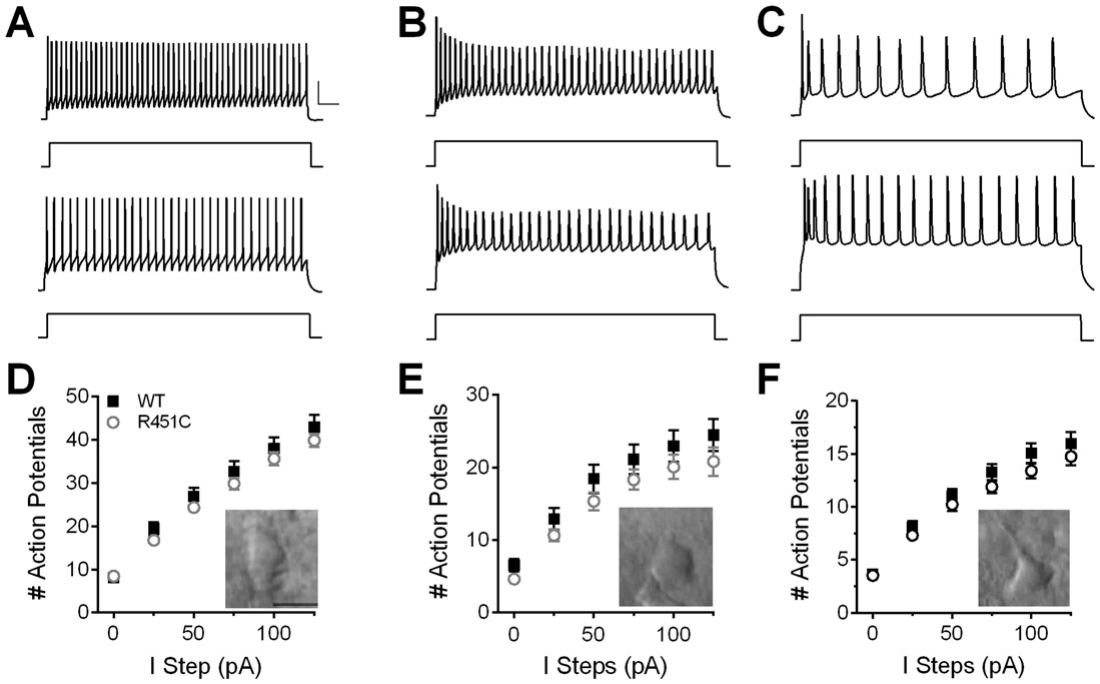
Firing properties of PV, SOM, and PYR neurons are not affected by the Nlgn3^R451C^ mutation. Raw traces of action potentials from (A) PV, (B) SOM, and (C) PYR neurons from WT (top) and Nlgn3^R451C^ (bottom) mice in response to a 125 pA step (represented by square pulse beneath each trace) above firing threshold. Scale bars: 25 mV; 50 ms. No difference is observed between Nlgn3^R451C^ and WT mice in the number of action potentials fired at 0–125 pA steps above firing threshold for PV (D), SOM (E), or PYR (F) neurons (PV: WT = 33, Nlgn3^R451C^ = 38; SOM: WT = 31, Nlgn3^R451C^ = 29; PYR: WT = 48, Nlgn3^R451C^ = 50). Inset: IR-DIC images taken with a 40X objective and 2X magnification of each respective cell type. Scale bar = 25 μm.

To determine whether the E/I imbalance at LII/III synapses is specific to either PV or SOM interneuron subtypes, heterozygous Nlgn3^R451C^ mice were crossed with mouse lines expressing EGFP in either PV or SOM interneurons. Intrinsic membrane properties and firing properties of each neuron were used to confirm neuron identity (see Fig 2 and Table 1). Resting membrane potential of SOM interneurons from Nlgn3^R451C^ mice was more hyperpolarized than from WT (*t* (58) = 2.81, *P* = 0.007), but no effect of genotype was found on resting membrane potential of PV or PYR neurons (PV: *t* (69) = 0.73, *P* = 0.467; PYR: *t* (103) = 0.64, *P* = 0.521). PV neurons from Nlgn3^R451C^ mice exhibited increased capacitance compared to WT (*t* (69) = 2.22, *P* = 0.03), but this effect was absent in SOM and PYR neurons. Input resistance was similar between WT and Nlgn3^R451C^ mice for all cell types (Table 1).

**Table 1:** Summary of intrinsic membrane properties of neurons from WT and Nlgn3^R51C^ mice. The resting membrane potential (RMP), capacitance (Cm), input resistance (R_Input_), and firing threshold of each cell was measured immediately following break-in. Data are represented as mean ± SEM.

Property	Cell Type	WT	Nlgn3^R451C^	Student’s t-test	Significance
RMP (mV)	PV	-61.69 ± 1.48, *n* = 33	-59.01 ± 3.16, *n* = 38	*t* (69) = 0.73	*P* = 0.467
	SOM	-54.94 ± 1.76, *n* = 31	-62.09 ± 1.84, *n* = 29	*t* (58) = 2.81	*P* = 0.007
	PYR	-65.78 ± 1.74, *n* = 53	-64.01 ± 2.14, *n* = 52	*t* (103) = 0.64	*P* = 0.521
Cm (pF)	PV	45.70 ± 4.06, *n* = 33	59.61 ± 4.65, *n* = 38	*t* (69) = 2.22	*P* = 0.030
	SOM	67.30 ± 4.95, *n* = 31	74.93 ± 5.22, *n* = 29	*t* (58) = 1.06	*P* = 0.293
	PYR	75.14 ± 3.19, *n* = 52	73.73 ± 3.79, *n* = 52	*t* (102) = 0.28	*P* = 0.777
R_Input_ (MΩ)	PV	194.1 ± 23.32, *n* = 33	170.1 ± 26.13, *n* = 38	*t* (69) = 0.68	*P* = 0.501
	SOM	328.9 ± 26.54, *n* = 31	360.2 ± 46.06, *n* = 29	*t* (58) = 0.60	*P* = 0.552
	PYR	261.3 ± 17.18, *n* = 52	236.3 ± 16.08, *n* = 52	*t* (102) = 1.06	*P* = 0.290
Threshold (pA)	PV	142.4 ± 21.33, *n* = 33	117.1 ± 12.30, *n* = 38	*t* (69) = 1.06	*P* = 0.292
	SOM	32.26 ± 5.34, *n* = 31	44.83 ± 5.46, *n* = 29	*t* (58) = 1.65	*P* = 0.105
	PYR	71.35 ± 7.02, *n* = 48	71.00 ± 4.97, *n* = 50	*t* (96) = 0.04	*P* = 0.967

In addition, firing threshold measured from resting membrane potential was not affected by the Nlgn3^R451C^ mutation in any cell type (Table 1). Action potential firing rate was determined for PV, SOM, and PYR neurons with a series of 25 pA current steps from threshold (Fig 2). Though the firing rate differed between cell types, there was no effect of genotype for PV (Genotype: *F*_(1,69)_ = 1.20, *P* = 0.278; Current Step: *F*_(5,345)_ = 309.8, *P* < 0.0001; Genotype X Current Step: *F*_(5,345)_ = 0.84, *P* = 0.525), SOM (Genotype: *F*_(1,54)_ = 1.86, *P* = 0.178; Current Step: *F*_(5,270)_ = 129, *P* < 0.0001; Genotype X Current Step: *F*_(5,270)_ = 0.28, *P* = 0.925), or PYR (Genotype: *F*_(1,93)_ = 1.32, *P* = 0.253; Current Step: *F*_(5,465)_ = 309, *P* < 0.0001; Genotype X Current Step: *F*_(5,465)_ = 1.17, *P* = 0.323) neurons. Therefore, changes in neuronal excitability at or near the soma are not likely to be responsible for increased inhibitory synaptic transmission in Nlgn3^R451C^ mice, suggesting an unknown synapse-level mechanism, instead.

### Bidirectional PV → PYR synaptic connections are unaffected by the Nlgn3^R451C^ mutation

**Fig 3:**
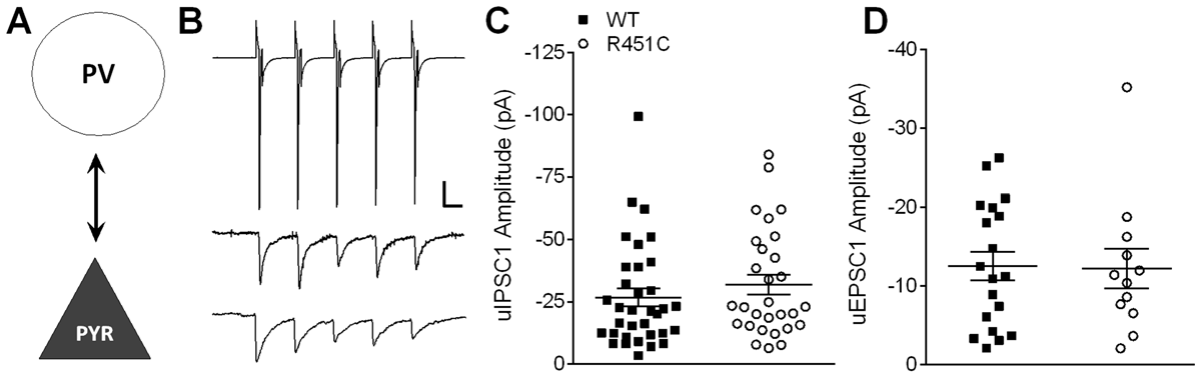
Bidirectional unitary postsynaptic responses at PV → PYR synapses are normal in Nlgn3^R451C^ mice. A) Simplified schematic of a PV → PYR synapse. B) Representative traces averaged from 20 consecutive sweeps (including failures). A short 20 Hz train of 5 action potentials is elicited from the presynaptic PV interneuron (in voltage clamp, top) and unitary IPSC (uIPSCs) are recorded from the postsynaptic pyramidal neuron (WT = middle; Nlgn3^R451C^ = bottom). Scale bars: 500 pA (PV), 25 pA (PYR); 25 ms. C) Scatter plot of uIPSC1 amplitude for each connected pair from WT (*n* = 34) and Nlgn3^R451C^ (*n* = 29). D) Scatter plot of uEPSC1 of connected PYR → PV pairs showing no difference in excitatory transmission onto PV interneurons between WT (*n* = 19) and Nlgn3^R451C^ (*n* = 12).

Next we used paired whole-cell recordings in voltage clamp between GFP-labeled interneurons and visually-identified, electrophysiologically confirmed pyramidal neurons (Fig 2) to determine whether the Nlgn3^R451C^ increase in inhibitory transmission is interneuron subtype-specific. Both cells were voltage-clamped at -55 mV and action potentials were generated in the presynaptic interneuron by a short train of 5 depolarizing voltage steps (25–75 mV) at 20 Hz. Corresponding unitary IPSCs (uIPSCs) were recorded in the postsynaptic pyramidal neuron (Fig 3A and 3B). Paired recordings are often bidirectional. Therefore, a subset of paired recordings were performed in the absence of NBQX and APV so that reciprocal excitatory and inhibitory transmission could be measured for each pair. Inhibitory-inhibitory connections were attempted, but occurred only rarely and thus could not be accurately quantified for the present study.

Connection frequency of PV → PYR pairs was high (WT = 0.72, *n* = 34/47 pairs, Nlgn3^R451C^ = 0.81, *n* = 29/36 pairs) and was not affected by the Nlgn3^R451C^ mutation (Mann-Whitney *U*-test, *U* = 776.5, *P* = 0.445). The mean amplitude of the first evoked uIPSC in the train was similar between WT and Nlgn3^R451C^ mice (Fig 3C; *t* (61) = 0.97, *P* = 0.338). As a means of measuring potential changes in short-term plasticity arising from the Nlgn3^R451C^ mutation, we compared the ratio of uIPSC5/uIPSC1 amplitudes since changes in this ratio may indicate changes in the probability of neurotransmitter release. However, the uIPSC5/uIPSC1 ratio was unchanged in PV → PYR pairs in Nlgn3^R451C^ compared to WT mice (*t* (61) = 0.38, *P* = 0.709), consistent with Tabuchi et al. [5] that found no effect of the Nlgn3^R451C^ mutation on inhibitory paired-pulse ratio.

Connection frequency of PYR → PV pairs was lower than that of PV → PYR pairs, but was not affected by the Nlgn3^R451C^ mutation (WT = 0.59, *n* = 17/29; Nlgn3^R451C^ = 0.39 *n* = 12/31; Mann-Whitney *U*-test, *U* = 479.5, *P* = 0.33). Mean uEPSC1 amplitude was not significantly different between WT and Nlgn3^R451C^ mice (Fig 3D; *t* (29) = 0.10, *P* = 0.918), nor was uEPSC5/uEPSC1 ratio (*t* (27) = 1.36, *P* = 0.186).

### Bidirectional SOM → PYR synaptic connections are unaffected by the Nlgn3^R451C^ mutation

**Fig 4:**
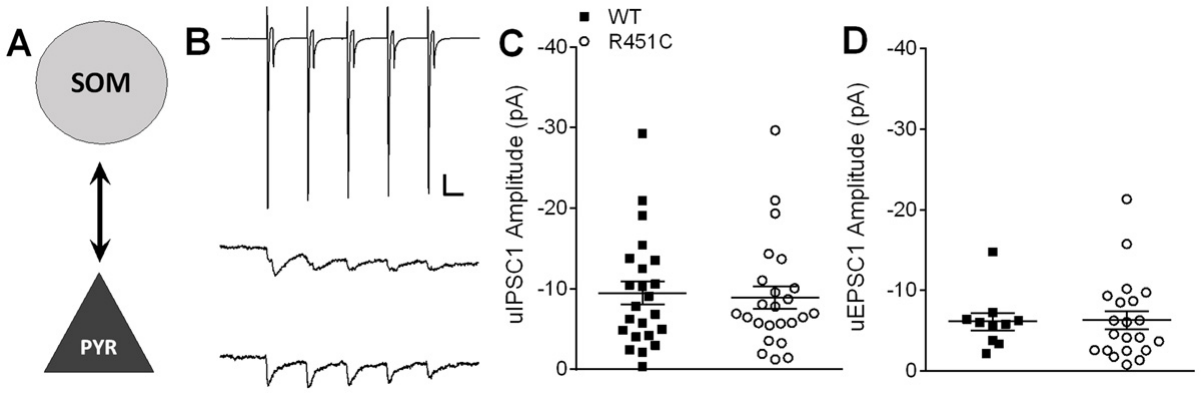
Bidirectional SOM → PYR unitary postsynaptic responses are unaffected by Nlgn3^R451C^ mutation. A) Simplified schematic of a SOM → PYR synapse. B) Representative traces averaged from 20 consecutive sweeps (including failures). A short 20 Hz train of 5 action potentials is elicited from the presynaptic SOM interneuron (in voltage clamp, top) and uIPSCs are recorded from the postsynaptic pyramidal neuron (WT = middle; Nlgn3^R451C^ = bottom). Scale bars: 600 pA (SOM), 10 pA (PYR); 25 ms. C) Scatter plot of uIPSC1 amplitude for each connected pair from WT (*n* = 23) and Nlgn3^R451C^ (*n* = 24). D) Scatter plot of uEPSC1 of connected PYR → SOM pairs showing no difference in excitatory transmission onto SOM interneurons between WT (*n* = 10) and Nlgn3^R451C^ (*n* = 21).

SOM → PYR pairs (Fig 4A and 4B) had a lower connection frequency (WT = 0.49 *n* = 23/47 pairs, Nlgn3^R451C^ = 0.40, *n* = 24/60 pairs) than PV → PYR pairs, but the difference in connection frequency between WT and Nlgn3^R451C^ SOM → PYR pairs was not significant (Mann-Whitney *U*-test, *U* = 1284, *P* = 0.433). SOM → PYR pairs also showed similar mean amplitudes and distributions of uIPSC1 between genotypes (Fig 4C. *t* (45) = 0.27, *P* = 0.789) and similar uIPSC5/uIPSC1 ratios (*t* (45) = 0.86, *P* = 0.395).

PYR → SOM connections also exhibited a relatively low connection frequency, but there was no effect of genotype (WT = 0.20 *n* = 10/49, Nlgn3^R451C^ = 0.39 *n* = 21/57; Mann-Whitney *U*-test, *U* = 1197, *P* = 0.089). There no effect of genotype on mean amplitude of uEPSC1 (Fig 4D. *t* (29) = 0.10, *P* = 0.918) or on uEPSC5/uEPSC1 ratio (*t* (29) = 0.44, *P* = 0.66). Taken together, our results suggest that PV and SOM synapses onto pyramidal neurons are not directly affected by the Nlgn3^R451C^ mutation in LII/III of somatosensory cortex, despite an overall increase in inhibitory synaptic tone and increased mIPSC frequency.

### PV and SOM cell number and density are not affected by the Nlgn3^R41C^ mutation

**Fig 5:**
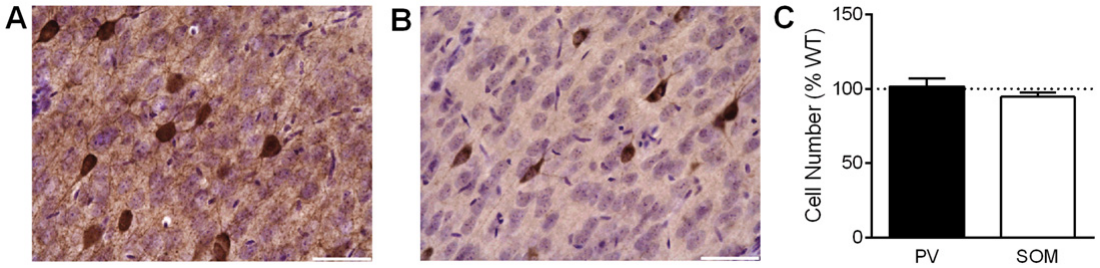
Number of PV and SOM interneurons is unaffected by the Nlgn3^R451C^ mutation. A) Representative image of DAB stained parvalbumin-positive cells with a Nissl counterstain in the somatosensory cortex. Scale bar = 50 μm. B) Representative image of DAB stained somatostatin-positive cells with a Nissl counterstain in the somatosensory cortex. Scale bar = 50 μm. C) The number of parvalbumin or somatostatin positive cells in the Nlgn3^R451C^ mutant somatosensory cortex counted using stereology and shown as percent of WT. WT = 10 mice, Nlgn3^R451C^ = 10 mice.

Previously, Tabuchi et al. [5] reported increased immunostaining for the inhibitory synaptic marker, VGAT, potentially indicating an increase in the number of inhibitory synapses. However, the number of symmetric synapses under electron microscopy, another measure of synapse number, was similar in WT and Nlgn3^R451C^ mice. Another possibility is that increased VGAT staining may reflect an increase in the number or density of interneurons. We immunostained for either parvalbumin (Fig 5A) or somatostatin (Fig 5B) and calculated the cell number and cell density across somatosensory cortex of WT and Nlgn3^R451C^ mice. There was no effect of genotype on cell number for parvalbumin (*t* (18) = 0.24, *P* = 0.813) or for somatostatin (*t* (18) = 0.96, *P* = 0.349) interneurons (Fig 5C), nor was there an effect of genotype on cell density (parvalbumin: *t* (10) = 0.60, *P* = 0.556; somatostatin: *t* (18) = 0.48, *P* = 0.636).

### Endocannabinoid signaling is impaired in Nlgn3^R451C^ mice

**Fig 6:**
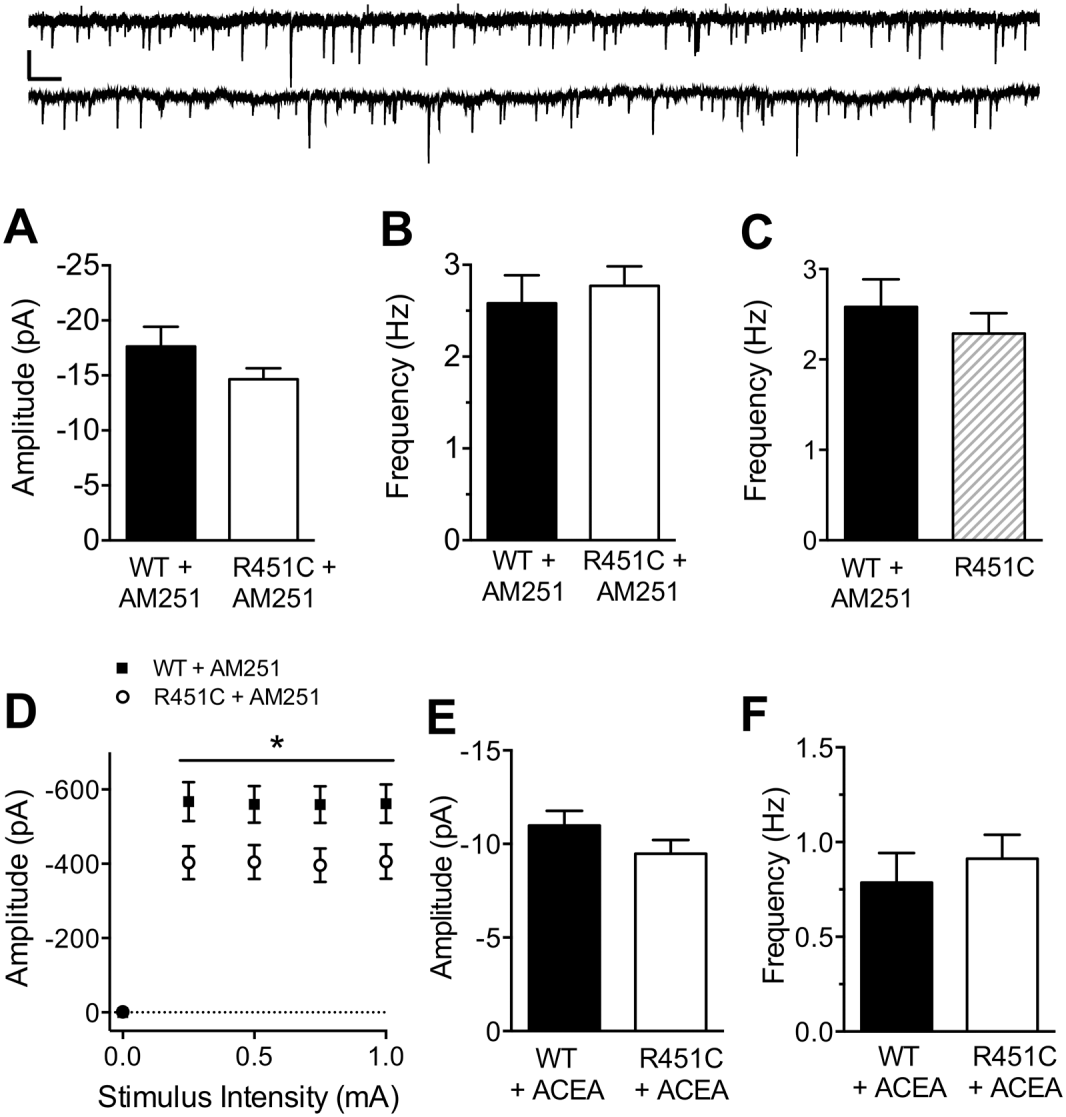
Tonic endocannabinoid signaling is decreased at cortical inhibitory synapses in Nlgn3^R451C^ mice. Mean amplitude (A) and frequency (B) of mIPSCs recorded in the presence of 1 μM TTX and 10 μM AM 251 from pyramidal neurons of WT (*n* = 23) and Nlgn3^R451C^ (*n* = 23) mice. Inset: 15 s raw traces from WT (top) and Nlgn3^R451C^ (bottom) pyramidal neurons. Scale bar = 25 pA, 0.5 s. C) The Nlgn3^R451C^-mediated increase in mIPSC frequency is occluded by bath application of AM 251 to neurons from WT mice (WT + AM 251 = 23, Nlgn3^R451C^ = 32). D) Input/output curves recorded in the presence of 10 μM AM 251 using the same range of stimulus intensities as in Fig 1C (WT = 22, Nlgn3^R451C^ = 23). Mean Amplitude (E) and frequency (F) of mIPSCs recorded in the presence of CB1 receptor agonist ACEA (10 μM; WT = 24, Nlgn3^R451C^ = 25).* P < 0.05.

eCBs have been well-studied in hippocampus, acting as retrograde signals that bind to presynaptic CB1 receptors, decreasing the probability of GABA release [10]. Tonic eCB signaling in the hippocampus is impaired in Nlgn3^R451C^ mice [8], but its role at LII/III synapses of somatosensory cortex is unknown. To determine whether the cortical increase in inhibitory transmission in Nlgn3^R451C^ mice is due to impaired tonic eCB signaling, we measured mIPSC amplitude and frequency in the presence of 1 μM TTX and 10 μM AM 251 (Fig 6A and 6B). mIPSC amplitude was not affected by AM 251 (WT: *t* (60) = 0.82, *P* = 0.418; Nlgn3^R451C^: *t* (53) = 1.81, *P* = 0.076), and mIPSC amplitude was not different between WT and Nlgn3^R451C^ in the presence of AM 251 (Fig 6A. *t* (44) = 1.46, *P* = 0.153).

When compared to mIPSCs from Fig 1A and 1B, AM 251 increased mIPSC frequency in neurons from WT (Student’s t-test. *t* (60) = 2.99, *P* = 0.004), but not in neurons from Nlgn3^R451C^ mice (Student’s t-test. *t* (53) = 1.51, *P* = 0.138). In the presence of AM 251, mean frequency (Fig 6B) was similar in neurons from WT and Nlgn3^R451C^ mice (Student’s t-test. *t* (44) = 0.51, *P* = 0.613), eliminating the Nlgn3^R451C^–mediated increase in mIPSC frequency compared to WT (Fig 1A). Finally, we find that blockade of CB1 receptors in pyramidal neurons of WT mice eliminates the difference between WT and Nlgn3^R451C^ mediated mIPSC frequency (Fig 6C. Student’s t-test. *t* (53) = 0.79, *P* = 0.432).

The effect of AM 251 on eIPSCs onto LII/III pyramidal neurons was also dramatic in WT mice, increasing eIPSC amplitude at all stimulus intensities (Two-way RM ANOVA. Genotype: *F*_(1,53)_ = 46.96, *P* < 0.001; Stimulus Intensity: *F*_(4,212)_ = 163.0, *P* < 0.001; Genotype X Stimulus Intensity: *F*_(4,212)_ = 42.16, *P* < 0.001). For example, average eIPSC amplitude at the strongest stimulus intensity (1 mA) is increased by 84.08% in the presence of AM 251 (No AM 251: -305.30 ± 34.86 pA, AM 251: -561.99 ± 50.81 pA; Student’s t-test. *t* (53) = 4.32, *P* < 0.0001). However, at inhibitory synapses onto pyramidal neurons from Nlgn3^R451C^ mice, the effect of AM 251 was only significant at 250 μA stimulus intensity (No AM 251: -171.71 ± 29.50 pA, AM 251: -402.85 ± 43.53 pA; Student’s t-test. *t* (51) = 4.55, *P* < 0.0001). There was no main effect of AM 251 when compared across all stimulus intensities (Two-way RM ANOVA. Genotype: *F*_(1,51)_ = 0.83, *P* = 0.366; Stimulus Intensity: *F*_(4,204)_ = 104.57, *P* < 0.001; Genotype X Stimulus Intensity: *F*_(4,204)_ = 11.92, *P* < 0.001; Bonferroni post-hoc analysis, *P* < 0.05), nor was there an effect of AM 251 at the strongest stimulus intensity, 1 mA (No AM 251: -469.00 ± 56.87 pA, AM 251: -405.91 ± 45.58 pA; Student’s t-test. *t* (51) = 0.83, *P* = 0.412). Interestingly, in the presence of AM 251, WT eIPSCs are stronger than Nlgn3^R451C^ eIPSCs at all stimulus intensities above 0 μA (Fig 6D. Two-way RM ANOVA. Genotype: *F*_(1,43)_ = 5.68, *P* = 0.022; Stimulus Intensity: *F*_(4,172)_ = 202.3, *P* < 0.001; Genotype X Stimulus Intensity: *F*_(4,172)_ = 5.70, *P* < 0.001).

Unlike AM 251 which had no effect on mIPSC amplitude (Fig 6A), bath application of the highly selective CB1 receptor agonist ACEA (10 μm) reduced mIPSC amplitude in both genotypes (WT: -19.42 ± 1.30 pA, WT + ACEA: -10.99 ± 0.79 pA, Student’s t-test.*t* (61) = 4.77, *P* < 0.0001; Nlgn3^R451C^: -17.86 ± 1.31 pA, Nlgn3^R451C^ + ACEA: -9.48 ± 0.74 pA, Student’s t-test. *t* (55) = 5.13, *P* < 0.0001). However, there remained no difference in mIPSC amplitude between WT and Nlgn3^R451C^ mice in the presence of ACEA (Fig 6E. Student’s t-test. *t* (47) = 1.40, *P* = 0.168). ACEA also reduced mIPSC frequency in both WT and Nlgn3^R451C^ mice (WT: 1.72 ± 0.13 Hz, WT + ACEA: 0.79 ± 0.20 Hz, Student’s t-test. *t* (61) = 4.57, *P* < 0.0001; Nlgn3^R451C^: 2.29 ± 0.22 Hz, Nlgn3^R451C^ + ACEA: 0.91 ± 0.13 Hz, Student’s t-test. *t* (55) = 4.95, *P* < 0.0001). There was no significant difference between WT and Nlgn3^R451C^ mIPSC frequency in the presence of ACEA (Fig 6F. Student’s t-test. *t* (47) = 0.63, *P* = 0.531). Also unlike AM 251, which normalized WT mIPSC frequency to Nlgn3^R451C^ levels, ACEA did not normalize Nlgn3^R451C^ mIPSC frequency to WT levels. Rather, ACEA depressed mIPSC frequency in Nlgn3^R451C^ mice below that of WT in the absence of agonist or antagonist (WT: 1.72 ± 0.13 Hz, Nlgn3^R451C^ + ACEA: 0.91 ± 0.13 Hz; Student’s t-test. *t* (62) = 4.57, *P* < 0.0001). These results support the hypothesis that impaired endocannabinoid signaling increases the probability of GABA release onto pyramidal neurons from Nlgn3^R451C^ mice, resulting in increased inhibitory synaptic transmission at LII/III cortical synapses.

## Discussion

Autism-associated cognitive deficits are thought to arise from abnormal local and global network connectivity, which affects sensory processing and behavior [17–19]. At the cellular level, connectivity is dependent on the proper E/I balance and is controlled, in part, by trans-synaptic interaction of Neuroligins with their presynaptic binding partners, Neurexins (NRX) [17, 20–23]. The increase in local inhibitory synaptic transmission at LII/III synapses in somatosensory cortex of Nlgn3^R451C^ mice may contribute to previously identified autism-associated behavioral deficits [5–7]. This study aimed to independently replicate these findings and to identify the specific inhibitory mechanisms affected by the Nlgn3^R451C^ mutation by targeting unitary connections between specific interneuron subtypes and pyramidal neurons, interneuron cell number and density, and endocannabinoid signaling.

Confirming the earlier studies of Tabuchi et al. [5], we found that inhibitory transmission onto pyramidal neurons in response to extracellular stimulation is increased in Nlgn3^R451C^ mice. Furthermore, this increase persists in the absence of action potential-dependent network activity, suggesting a local alteration at the level of individual synapses. Inhibitory transmission in LII/III is highly dependent on two major interneuron subtypes that form functionally distinct networks to tightly regulate synaptic activity in somatosensory cortex: Fast-spiking PV interneurons and low-threshold SOM interneurons [14, 24, 25]. Each interneuron subtype is distinguishable by its gene expression [11, 26] and intrinsic firing pattern [27].

Other autism-related mouse models have been shown to have interneuron subtype-specific effects including the Nlgn2 knockout mouse with impaired PV inhibitory transmission [9] and the Fmr1 mouse model of Fragile X syndrome with impairments in both PV [28] and SOM inhibitory networks [29]. In addition, PV → pyramidal connections are weaker in the hippocampus of Nlgn3^R451C^ mice, leading to a local increase in excitatory transmission [8]. In cortex, however, we found no difference in cell-to-cell unitary synaptic strength between PV or SOM interneurons and LII/III pyramidal neurons.

Theoretically, increased inhibitory tone of somatosensory cortex of Nlgn3^R451C^ mice may be due to a larger number of inhibitory synapses, consistent with enhanced staining for the inhibitory synapse marker VGAT [5, 30]. This increase in inhibitory synapse number could arise from multiple potential mechanisms, including increased inhibitory synaptic connection frequency between neurons, increased strength of existing inhibitory synaptic connections, or increased numbers of inhibitory interneurons. Our data demonstrating no change in uIPSCs from SOM and PV interneurons argue against an increase in number or strength of individual PV → PYR or SOM → PYR inhibitory synapses. In addition, we found no difference in cell number or density of PV-expressing or SOM-expressing interneurons using immunochemistry and stereology in somatosensory cortex of WT and Nlgn3^R451C^ mice. Thus, we can effectively rule out a contribution of either PV or SOM interneurons to increased inhibitory synaptic function in LII/III of somatosensory cortex in Nlgn3^R451C^ mutants. While these are the predominant inhibitory neurons of this region, multiple other inhibitory interneuron subtypes may contribute.

Foldy et al. [8] have previously demonstrated that impaired tonic, but not phasic eCB signaling leads to disinhibition of excitation in hippocampus of Nlgn3^R451C^ mice, but it is unknown whether altered eCB signaling plays a role in increased inhibitory transmission at cortical synapses. eCBs decrease the probability of neurotransmitter release by binding to CB1 receptors, which in turn, limit Ca^2+^ influx through N-type (Cav2.2) channels [10]. A decrease in tonic eCB signaling at inhibitory synapses onto pyramidal neurons in cortex would result in disinhibition of GABA release, accounting for the increased frequency of mIPSCs and amplitude of extracellularly-evoked eIPSCs of Nlgn3^R451C^ somatosensory cortex. Indeed, our results using the CB1 receptor antagonist AM 251 suggest that a decrease in tonic eCB signaling is the driving force behind the cortical increase in inhibitory transmission in Nlgn3^R451C^ mice.

Consistent with Nlgn3^R451C^ mutation leading to decreased tonic eCB signaling, AM 251 blockade of eCB signaling completely eliminates the increase in inhibitory mIPSC frequency in Nlgn3^R451C^ mice compared to WT. If tonic eCB signaling inhibition alone were responsible for the increased inhibition in Nlgn3^R451C^ cortex, then AM 251 would be expected to normalize I/O curves for WT up to the level of Nlgn3^R451C^ mice. Consistent with this hypothesis, AM 251 had a much more dramatic effect on WT inhibitory I/O curves compared to that of Nlgn3^R451C^ inhibitory I/O curves. In addition, in the presence of AM 251, inhibitory I/O curves were greater in WT compared to Nlgn3^R451C^ mice, indicating a partial role for impaired phasic eCB signaling in Nlgn3^R451C^ mice in addition to the effect on tonic eCB signaling. Further attempts were made to re-define I/O curves in the presence of AM 251, but stimulation resulted in an “all or none” response in neurons of both genotypes.

The effects of AM 251 on mIPSC frequency and amplitude are limited to endogenously activated CB1 receptors. We repeated the mIPSC experiment in the presence of CB1 agonist ACEA, which theoretically activates most CB1 receptors on the cell surface. As expected, ACEA decreases mIPSC frequency in WT and Nlgn3^R451C^ mice and, like AM 251, eliminates the increased mIPSC frequency characteristic of Nlgn3^R451C^ mice compared to WT. Furthermore, ACEA reduces mIPSC amplitude by half in both genotypes, with no difference between genotypes. These experiments yield two conclusions: 1) Not all CB1 receptors are tonically active under normal recording conditions and 2) Expression of functional CB1 receptors is similar in WT and Nlgn3^R451C^ mice.

How does a single substitution mutation in the Nlgn3 gene cause altered endocannabinoid signaling and increased inhibition, yet complete loss of Nlgn3 has no effect on either excitatory or inhibitory synaptic transmission in cortex? The answer remains unclear and is confounded by very different effects of the Nlgn3^R451C^ mutation and Nlgn3 knockout in hippocampus [5, 8]. Here we have narrowed the target mechanisms to altered eCB signaling, at inhibitory synapses that do not involve Parv or Som interneurons. Based on recent literature, the most likely candidate interneuron for altered eCB signaling is the cholecystokinin (CCK) interneuron subtype [8]. Altered eCB signaling at CCK synapses onto CA1 pyramidal neurons of the hippocampus was found in both Nlgn3^R451C^ and Nlgn3 KO mice [8], and CB1 receptor expression is found in CCK interneurons, but not Parv interneurons [31].

Another issue for further study is the contribution of phasic versus tonic endocannabinoid activity. We have shown that blockade of endogenous tonic CB1 signaling eliminates the two major electophysiological phenotypes in the cortex of the Nlgn3^R451C^ mouse: increased mIPSC frequency and increased inhibitory I/O curve. That AM 251 not only eliminates the increase in eIPSC amplitude between Nlgn3^R451C^ and WT mice, but elicits an even greater response in neurons from WT mice suggests that there is greater activity-dependent endocannabinoid signaling, in addition to tonic signaling, in WT mice. One possible explanation is that tonic eCB activity is a presynaptic function, whereas phasic eCB function is postsynaptic. Another is that the Nlgn3^R451C^ mutation also causes impairment of the postsynaptic scaffold, thus indirectly preventing the necessary rise in intracellular calcium needed for activity-dependent eCB retrograde signaling [32–35].

The contrasting effects of the Nlgn3^R451C^ mutation on E/I balance in cortex and hippocampus have been a major conundrum since their discovery by Etherton et al. [6]. Here, we identify impaired tonic eCB signaling as a common mechanism of Nlgn3^R451C^-induced E/I imbalance in cortex, consistent with that identified in hippocampus by Foldy et al [8]. Thus tonic eCB signaling is a common mechanism affected by the Nlgn3^R451C^ mutation, and is crucial for maintaining proper E/I balance in a region-specific manner. Further study is necessary to determine the relative contributions of tonic and phasic eCB signaling at cortical synapses, as well as the specific interneuron subtype affected by altered eCB signaling in cortex of both Nlgn3^R451C^ and Nlgn3 KO mice.
